# La gangrène de Fournier compliquant un empalement

**DOI:** 10.11604/pamj.2015.21.250.7601

**Published:** 2015-08-06

**Authors:** Ammar Mahmoudi, Abdelaziz Hamdi

**Affiliations:** 1 Service de Chirurgie Générale et Digestive, CHU Fattouma Bourguiba de Monastir, Monastir, Tunisie

**Keywords:** Empalement ano-rectal, gangrène de Fournier, fasciite nécrosante, réanimation, antibiothérapie, chirurgie, anorectal impalement, Fournier gangrene, necrotizing fasciitis, reanimation, antibiotherapy, surgery

## Image en medicine

La gangrène de Fournier (GF) est une fasciite bactérienne nécrosante rapidement progressive du périnée et des organes génitaux externes. Elle est secondaire à une infection polymicrobienne. Elle représente une urgence médico-chirurgicale nécessitant une prise en charge multidisciplinaire. L’étiologie est identifiée dans 95% des cas. La source de l'infection est cutanée, urogénitale ou colorectale. Des facteurs favorisants, comme l’âge, le diabète et l'immunodépression, sont souvent présents. La clinique est fulminante. La mortalité reste élevée, de l'ordre de 20 à 80%, souvent en raison du retard de diagnostic et de prise en charge. Nous rapportons l'observation d'un patient de 44 ans sans antécédents, maçon, qui avait présenté un empalement anorectal accidentel sur une tige de fer à béton. Il a été vu dans un hôpital régional où il a eu simplement des soins locaux. Trois jours plus tard, il consulte pour douleur périnéale. Il était fébrile à 40°C, son état général était très altéré avec tendance au collapsus. Il existait un érythème périnéal, périanal et du scrotum avec des crépitations neigeuses à la palpation. La plaie périanale faisait sourdre du pus de couleur jaune verdâtre et d'odeur nauséabonde. Le diagnostic de GF a été retenu. Une rééquilibration hydro-électrolytique, avec une antibiothérapie à large spectre suivie rapidement par un débridement chirurgical agressif ont été réalisées. A l'exploration, il exsistait une plaie rectale sus-anale qui a été suturée et protégée par une colostomie qui sera fermée six mois plus tard. Des soins locaux ont permis une cicatrisation dirigée obtenue au bout de 3 mois.

**Figure 1:**
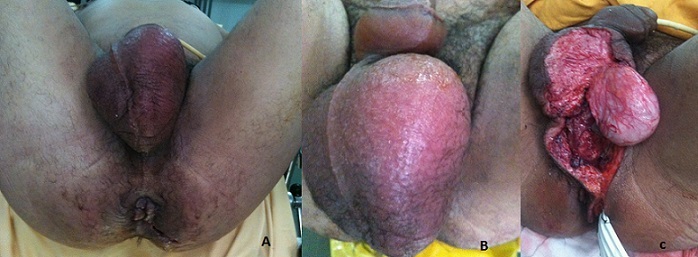
(A) vue opératoire du patient en position de la taille montrant l’érythème et l’œdème périnéal, périanal et du scrotum. La palpation trouvait des crépitations neigeuses. La plaie périanale traumatique à 4 H faisait sourdre du pus de couleur jaune verdâtre et d'odeur nauséabonde; (B) vue opératoire du patient en position de la taille montrant l’érythème et l’œdème du scrotum et de la verge. La palpation trouvait des crépitations neigeuses; (C) vue opératoire montrant l’état local satisfaisant après le premier débridement agressif avec mise à nue du testicule gauche ainsi que le drainage par lame ondulée

